# Identification of biomarkers in common chronic lung diseases by co-expression networks and drug-target interactions analysis

**DOI:** 10.1186/s10020-019-0135-9

**Published:** 2020-01-17

**Authors:** Mazaher Maghsoudloo, Sadegh Azimzadeh Jamalkandi, Ali Najafi, Ali Masoudi-Nejad

**Affiliations:** 1 Laboratory of Systems Biology and Bioinformatics (LBB), Department of Bioinformatics, Kish International Campus, University of Tehran, Kish Island, Iran; 2 Laboratory of Systems Biology and Bioinformatics (LBB), Institute of Biochemistry and Biophysics, University of Tehran, Tehran, Iran; 3 Chemical Injuries Research Center, Systems Biology and Poisonings Institute, Tehran, Iran; 4 Molecular Biology Research Center, Systems Biology and Poisonings Institute, Tehran, Iran

**Keywords:** Chronic lung diseases, Asthma, COPD, IPF, Gene co-expression network, Module, Consensus, Biomarker, Drug-target

## Abstract

**Background:**

asthma, chronic obstructive pulmonary disease (COPD), and idiopathic pulmonary fibrosis (IPF) are three serious pulmonary diseases that contain common and unique characteristics. Therefore, the identification of biomarkers that differentiate these diseases is of importance for preventing misdiagnosis. In this regard, the present study aimed to identify the disorders at the early stages, based on lung transcriptomics data and drug-target interactions.

**Methods:**

To this end, the differentially expressed genes were found in each disease. Then, WGCNA was utilized to find specific and consensus gene modules among the three diseases. Finally, the disease-disease similarity was analyzed, followed by determining candidate drug-target interactions.

**Results:**

The results confirmed that the asthma lung transcriptome was more similar to COPD than IPF. In addition, the biomarkers were found in each disease and thus were proposed for further clinical validations. These genes included RBM42, STX5, and TRIM41 in asthma, CYP27A1, GM2A, LGALS9, SPI1, and NLRC4 in COPD, ATF3, PPP1R15A, ZFP36, SOCS3, NAMPT, and GADD45B in IPF, LRRC48 and CETN2 in asthma-COPD, COL15A1, GIMAP6, and JAM2 in asthma-IPF and LMO7, TSPAN13, LAMA3, and ANXA3 in COPD-IPF. Finally, analyzing drug-target networks suggested anti-inflammatory candidate drugs for treating the above mentioned diseases.

**Conclusion:**

In general, the results revealed the unique and common biomarkers among three chronic lung diseases. Eventually, some drugs were suggested for treatment purposes.

## Introduction

Lungs are considered as vital and vulnerable parts of the respiratory system and play critical roles in the body (Soriano et al. 2017). Several disorders affect some parts of the respiratory system and reduce lung functions, including chronic lung disease, which is among the most common type of diseases. Based on previous evidence, hundreds of millions of people suffer from this disease worldwide, and its prevalence is increasing among children and the elderly (Chuchalin et al. 2014). Nowadays, lung disease is one of the leading causes of mortality, claiming the lives of at least four million people annually in the world (Soriano et al. 2017; Chuchalin et al. 2014). Some of the most common chronic lung diseases include chronic obstructive pulmonary disease (COPD), asthma, and idiopathic pulmonary fibrosis (IPF), which have shared features and distinctions. The three above mentioned lung diseases share characteristics such as chronic, progressive, reduced lung function, and inflammation (Soriano et al. 2017; Vestbo et al. 2013; Duck et al. 2015; García-Sancho et al. 2011). On the other hand, smoking, air pollution, infection, and genetic parameters are among the risk factors that affect the development of these diseases (Postma and Rabe 2015; Nie et al. 2017; O’Donnell et al. 2016). In addition, cough, dyspnea, chest tightness, shortness of breath, and mucus production are known as some of the shared clinical symptoms in such diseases (Vestbo et al. 2013; Postma and Rabe 2015; Reddel et al. 2015).

COPD and asthma are known by the limitations in the airways, inflammation, airway obstruction, and bronchial interactions (Soriano et al. 2017; Vestbo et al. 2013; Postma and Rabe 2015) while alveoli are damaged and injured in IPF. Therefore, asthma and COPD are considered as lung diseases that affect the airways while IPF affects the interstitium (Soriano et al. 2017; Vestbo et al. 2013; O’Donnell et al. 2016). Furthermore, COPD and IPF are among the irreversible diseases whereas asthma is reversible (Duck et al. 2015; García-Sancho et al. 2011). In addition, COPD and IPF are more common among the elderly while asthma is prevalent in the elderly and children (Soriano et al. 2017; García-Sancho et al. 2011; O’Donnell et al. 2016).

Nearly 329 million people (approximately 5% of the world population) are struggling with COPD. The incidence rate has indicated an increase of 44.2% from 1990 to 2015 (Soriano et al. 2017). According to the World Health Organization (WHO), COPD is reported as the third leading cause of death worldwide (Nie et al. 2017). In 2015, about 3.2 million people died because of COPD and mortality rates increased by 11.6% compared to 1990 (Soriano et al. 2017), representing an increase in COPD in the world. It is noteworthy that the global prevalence of asthma is estimated to be 300 million (Peters et al. 2006) and between 1 and 21% among adults (Reddel et al. 2015). Approximately, 0.4 million people died due to this disease in 2015 (Soriano et al. 2017). Moreover, the number of people with asthma is increasing each year, which is mostly occurs among children (Peters et al. 2006). However, the prevalence of IPF is approximately 23 in every 100,000 people (Duck et al. 2015). It is worth noting that most IPF patients have a history of smoking and the incidence of this disease in males is higher compared to females (O’Donnell et al. 2016).

The clinical symptoms of these diseases are similar in the early stages, and thus they cannot be differentiated clearly. Accordingly, the systems biology approach is essential for understanding the molecular mechanisms of these three diseases. Previous studies have only examined the overlap mechanism between the two diseases. Therefore, the present study sought to investigate the mechanisms of COPD, asthma, and IPF simultaneously using systems biology approaches. To this end, disease-specific and consensus candidate modules were identified using the co-expression networks, and then ten hub differentially expressed genes were introduced in each case. Eventually, candidate drug-target interactions were predicted for these diseases.

## Methods

### Dataset selection and differential gene expression analysis

The transcriptomic datasets were retrieved from the NCBI Gene Expression Omnibus database (GEO) (Edgar et al. 2002), including lung tissue biopsies in COPD, asthma, and IPF diseases (Table 1). Raw data were pre-processed using the Limma package in R (Smyth 2005), followed by performing the quantile normalization and quality control, as well as identifying and removing the noisy data and outliers by hierarchical clustering. Additionally, the differentially expressed genes (DEGs) were identified using the EBayes method in Limma (Smyth 2004). Finally, the DEGs in each disease (adjusted *P* value< 0.05) were estimated based on healthy and patient states for the samples (COPD vs. healthy, IPF vs. healthy, and asthma vs. healthy). The mean of expression was used for multiple probes mapping to the same gene (Liu et al. 2016).

**Table 1:** Characteristics of Selected Microarray Data Series

Datasets	Disease	Grouping		Sample	Platform	Expression Array	Ref.
		Case	Control				
GSE47460	COPD^a^	71	91	Lung biopsy	GPL14550	Agilent-028004 SurePrint G3 Human GE 8x60K Microarray	(Peng et al. 2016)
GSE47460	IPF	122	91	Lung biopsy	GPL14550	Agilent-028004 SurePrint G3 Human GE 8x60K Microarray	(Peng et al. 2016)
GSE23611	asthma	27	13	Lung biopsy	GPL6480	Agilent-014850 Whole Human Genome Microarray 4x44K G4112F	(Choy et al. 2011)

^a^GOLD stage II

### Disease-disease expression similarity analysis

**Fig. 1:**
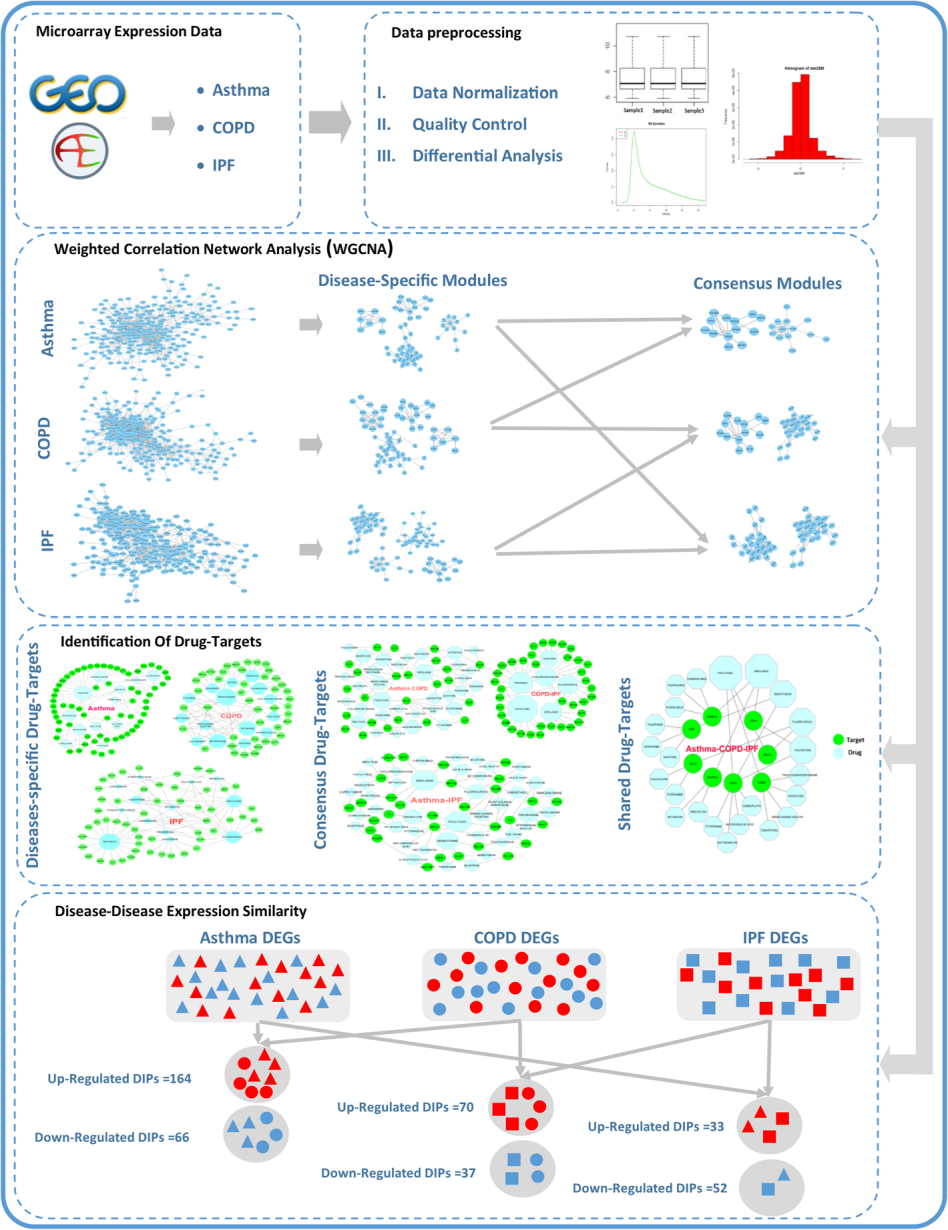
Workflow of data preparation and analysis

In order to survey the common mechanisms among three diseases, the DEGs that have identical expression levels rather than healthy controls were identified (i.e., the up/down-regulated DEGs between two or among three diseases). The gene list of shared DEGs between the two diseases (three states) and among the three diseases was used for further investigations (Fig. 1).

### Co-expression network construction and disease-specific module identification

According to the study by Horvath and Dong (Horvath and Dong 2008), the signed weighted gene co-expression network (WGCN) was firstly constructed considering biweight midcorrelation (Song et al. 2012) between the disease-specific DEGs (Huang et al. 2017; Chen et al. 2017; Motieghader et al. 2017). Next, the modules were identified.

Similarly, the optimal β parameter in the weighted gene co-expression network analysis (WGCNA) was calculated using the pickSoftThreshold function. Then, disease-specific adjacency matrices were generated utilizing the selected β threshold and were transformed to the Topological Overlap Matrix (TOM) using the TOMsimilarity function (Yip and Horvath 2007) to represent the correlation between the overlap of the neighbors in the constructed biological networks (Ravasz et al. 2002). Then, the hierarchical average linkage clustering of 1-TOM (dissTOM) was applied to extract the modules, namely, a set of genes that behave in the same functional manner (Liu et al. 2016). In addition, the function of cutreeDynamic (minModuleSize =10) was used to determine the optimal cutset to identify the functional modules for each disease. Based on their gene expression profiles (Zhang and Horvath 2005), similar modules were combined by the eigengene through the moduleEigengenes function (Langfelder and Horvath 2008).

Module eigengene (ME) is defined as the first principal component of the gene expression in each module. In the present study, the MEs were combined using the mergeCloseModules function based on their similarity in the profile of the gene expression. This function uses a cutHeight parameter to determine the correlation among the MEs. The parameter was set to 0.15 in this study, and modules with a correlation ≥0.85 (1-cutHeight) were merged accordingly. Finally, three subsequent analyses were used to identify the most important modules for each disease as follows (Fig. 1).

- *Analysis of module-trait association*: It was performed based on the disease state to specify the correlation between the modules and the disease states. Modules with a correlation of ≥|0.5| were then selected.
- *Analysis of module membership and gene significance (MM-GS)*: The correlation between gene expression profile in the modules and the disease states, as well as the correlation between the MEs and the gene expression were referred to as Gene Significance (GS) and Module Membership (MM), respectively. Modules with a *P* < 0.05 were filtered in the MM-GS analysis for each disease.
- *Analysis of determining the genes associated with the disease*: The DisGeNET (version 5.0) online database was used to determine the number of genes associated with each disease within the significant modules that were obtained in previous steps (a and b) (Piñero et al. 2016). This database contains 561,119 gene-disease relationships in humans. Eventually, the Chi-square statistical method was utilized to assess the significance of module-genes in each disease-gene category in DisGeNET (Alaei et al. 2018).

Afterward, the top 10 hub genes were identified in disease-specific modules among the three diseases. Moreover, novel hub DEGs involved in the processes and pathways associated with the diseases were extracted through literature mining (Najafi et al. 2014). Finally, the reported DEGs in DisGeNET and novel disease-DEGs were illustrated in the modules.

### Consensus network analysis (CNA)

Based on ref. (Langfelder and Horvath 2007), the CNA was performed to identify Consensus Modules (CMs). Three consensus networks (CNs) were constructed based on the shared DEGs among the diseases. First, the list of the shared DEGs was employed to retrieve the expression data for each disease (two datasets in each analysis). Then, six extracted expression data were fed into the CNA, followed by separately setting the parameters for each of the three CNAs. TOM matrices with a precision of 0.95 were aligned, given that TOM of the two datasets in each analysis might have different statistical properties (Langfelder and Horvath 2007). Next, to form a CN between the two diseases (i.e., COPD vs. IPF), TOM-aligned matrices in two diseases were combined using the quantile function as follows:

$$ Consensus\_ TOM\left(i,j\right)=\mathrm{pquantile}\left\{{TOM}_{Disease1}\left(i,j\right),{TOM}_{Disease2}\left(i,j\right), prob=0.1\right\} $$

The stringency of CN identification relies on the *prob* parameter of the function. In other words, it is more stringent when it tends to zero (Langfelder and Horvath 2007). The cutreeDynamic function uses 1-Consensus_TOM for extracting the CMs in the CNAs. The MEs were computed and used to combine similar CMs with a correlation of ≥0.85. Afterward, the Module-Trait Association was used to identify the most important modules with a correlation of ≥|0.5|. Then, the top 10 hub genes were identified in the CMs. Moreover, literature mining was conducted to find the novel hub DEGs that were associated with the diseases (Najafi et al. 2014). Finally, the reported DEGs in DisGeNET and novel disease-DEGs were represented in the CMs.

The CNA among the three diseases was constructed based on their shared DEGs as well. The CMs among the three diseases were extracted and used for the module-trait association analysis to determine important CMs (Fig. 1).

### Enrichment analysis

Gene enrichment analysis was applied to functionally assess the identified modules in Gene Ontology (GO) and pathway databases, including the Kyoto Encyclopedia of Genes and Genomes (KEGG), Biocarta, and Reactome via the Enrichr (adjusted *P* < 0.05) (Kuleshov et al. 2016). Therefore, the enrichment analysis was used for functional evaluation of the identified modules in each disease and the CMs among the diseases.

### Identification of candidate drug-targets

Drug Gene Interaction Database (DGIdb, version 3.0 updated on 25-01-2018) was utilized to identify the candidate drugs in the studied lung diseases (Cotto et al. 2017). The list of DEGs (i.e., disease-specific DEGs, and the shared DEGs between two/among three diseases) was used to retrieve drug-DEG interactions from the DGIdb and to construct drug-target networks. Network visualization was performed using open-source Cytoscape software, version 3.6.0 (Shannon et al. 2003) as well (Fig. 1).

## Results

### Dataset selection and differential gene expression analysis

**Fig. 2:**
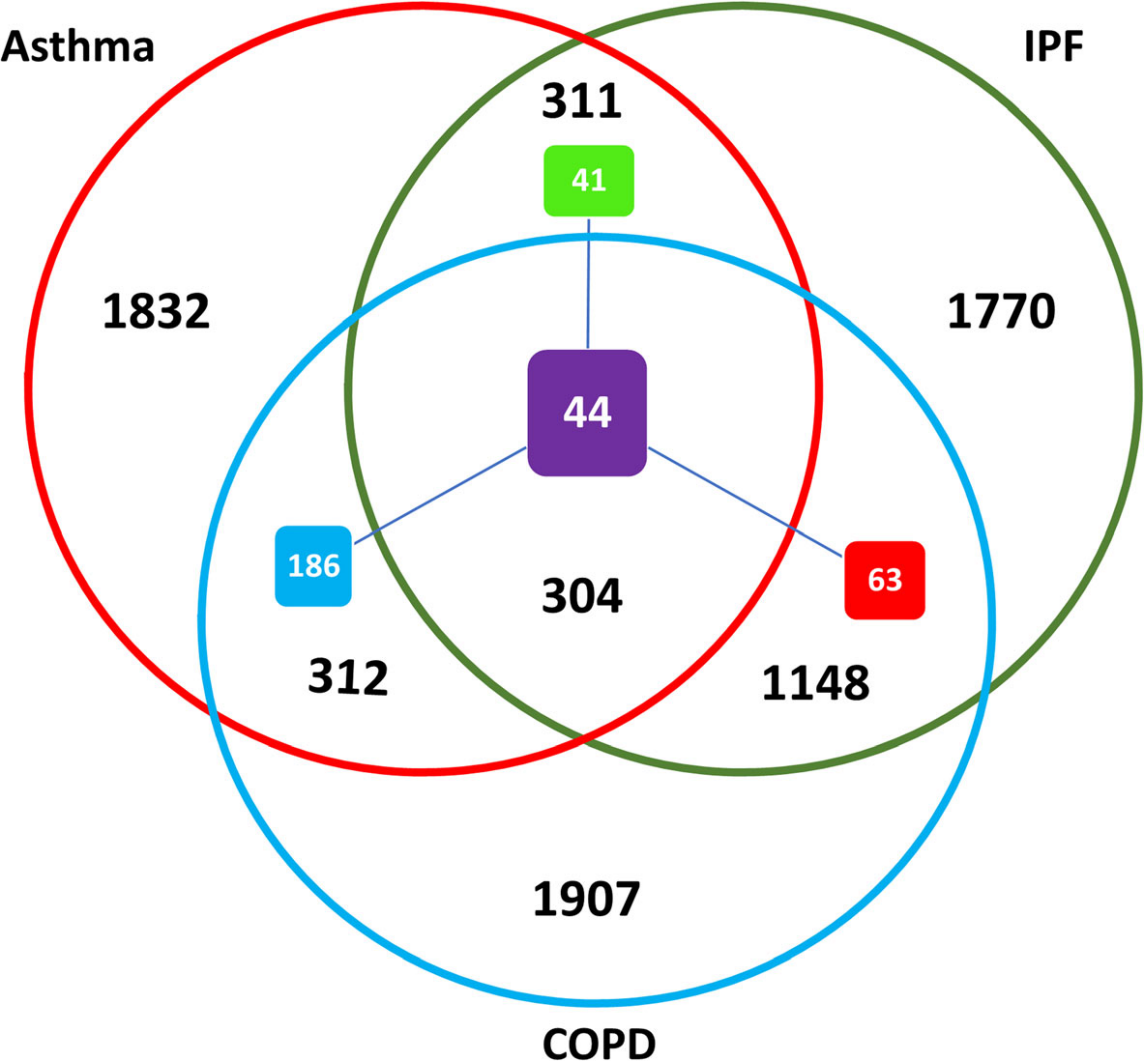
Venn diagram illustrating the specific and shared DEGs and DIPs among three diseases. The red, blue, and green circles represent asthma, COPD, and IPF DEGs, respectively. The violet and blue, as well as the violet and green squares demonstrate the asthma-COPD and asthma-IPF DIPs, respectively. Finally, the violet and red squares represent COPD-IPF DIPs and the violet square indicates the DIPs among three diseases

Two microarray gene expression datasets of GSE47460 (GPL14550) and GSE23611 (GPL6480) were used in this study. After data normalization and quality control, 34 COPD samples (only GOLD stage II, Moderate COPD patients) and 32 healthy controls, as well as 118 IPF samples and 86 healthy controls from the GSE47460 dataset were utilized to determine the DEGs. In addition, 26 samples from asthmatic patients and 7 healthy controls were selected from GSE23611. The results of differential analyses are displayed in Fig. 2. Totally, 2759, 3671, and 3533 were found as the DEGs between asthma, COPD, and IPF versus healthy controls, respectively.

### Analysis of disease-disease expression similarity

The DEGs with a matching up/down-regulated pattern between two or among three diseases were nominated as the DEGs with Identical Pattern (DIPs) to assess disease-disease expression similarity. The results of the three pairwise comparisons revealed 616, 615, 1452, and 304 shared DEGs between asthma-COPD, asthma-IPF, COPD-IPF, and asthma-COPD-IPF, respectively. Table 2 presents the number of up/down-regulated DIPs and the list of DIPs and shared DEGs are provided in Additional file 1.

**Table 2:** The Number of DIPs and Shared DEGs among the Diseases

Type	asthma-COPD	COPD-IPF	asthma-IPF	asthma-COPD-IPF
Shared DEGs	616	1452	615	304
Shared DIPs	230	107	85	44
Up-regulated DIPs	164	70	33	27
Down-regulated DIPs	66	37	52	17

Based on the results, there are more DIPs between asthma and COPD compared to COPD and IPF, as well as asthma and IPF. However, there are more DIPs between COPD and IPF compared to asthma and COPD or asthma and IPF.

### Co-expression network construction and disease-specific module identification

**Fig. 3:**
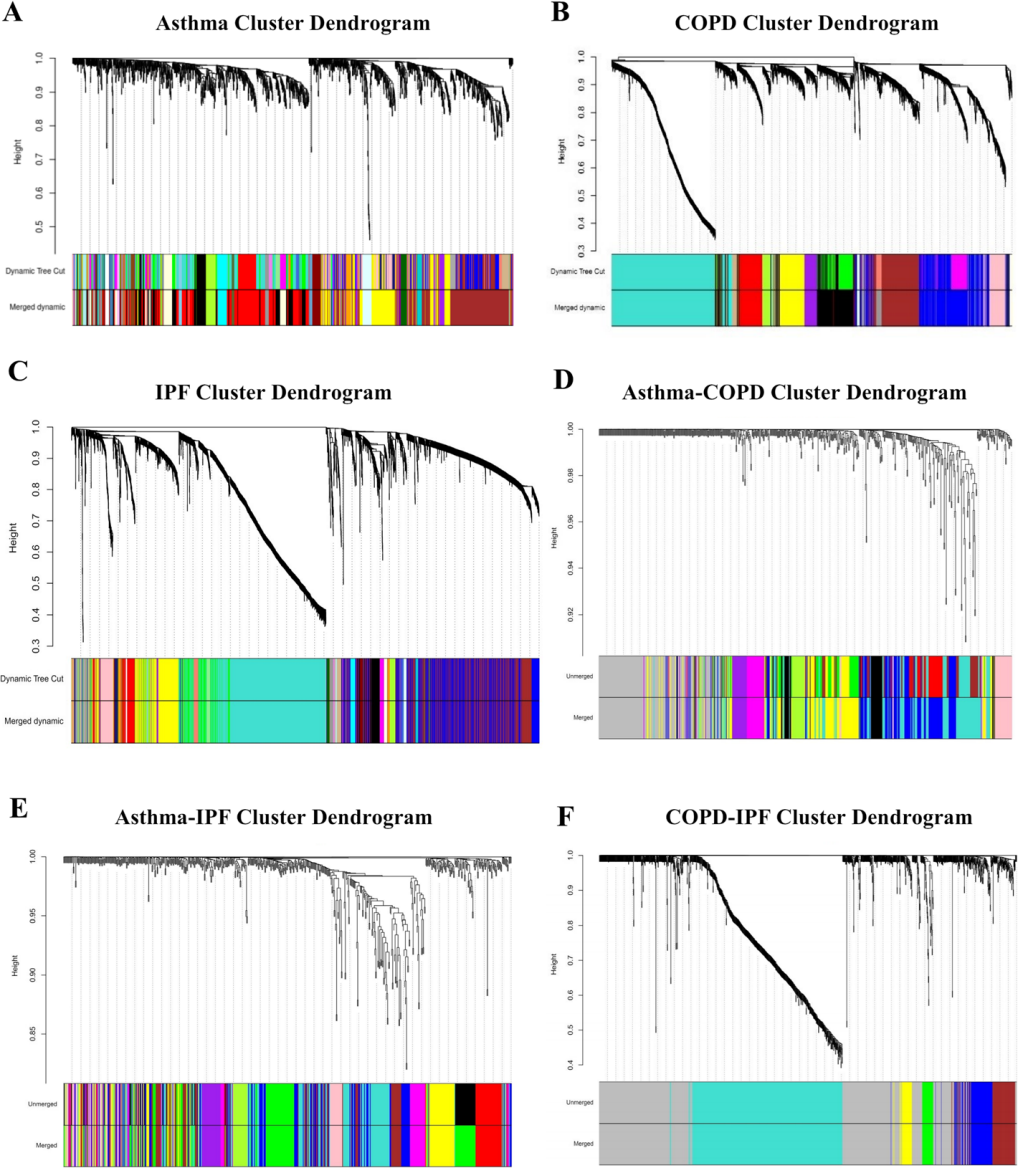
The clustering dendrogram of genes. **a** Gene clustering situation in asthma data, **b** Gene clustering situation in COPD data, **c** Gene clustering situation in IPF data, **d** Gene clustering situation in the asthma-COPD data, **e** Gene clustering situation in the asthma-IPF data, and **f** Gene clustering situation in COPD-IPF data

**Fig. 4:**
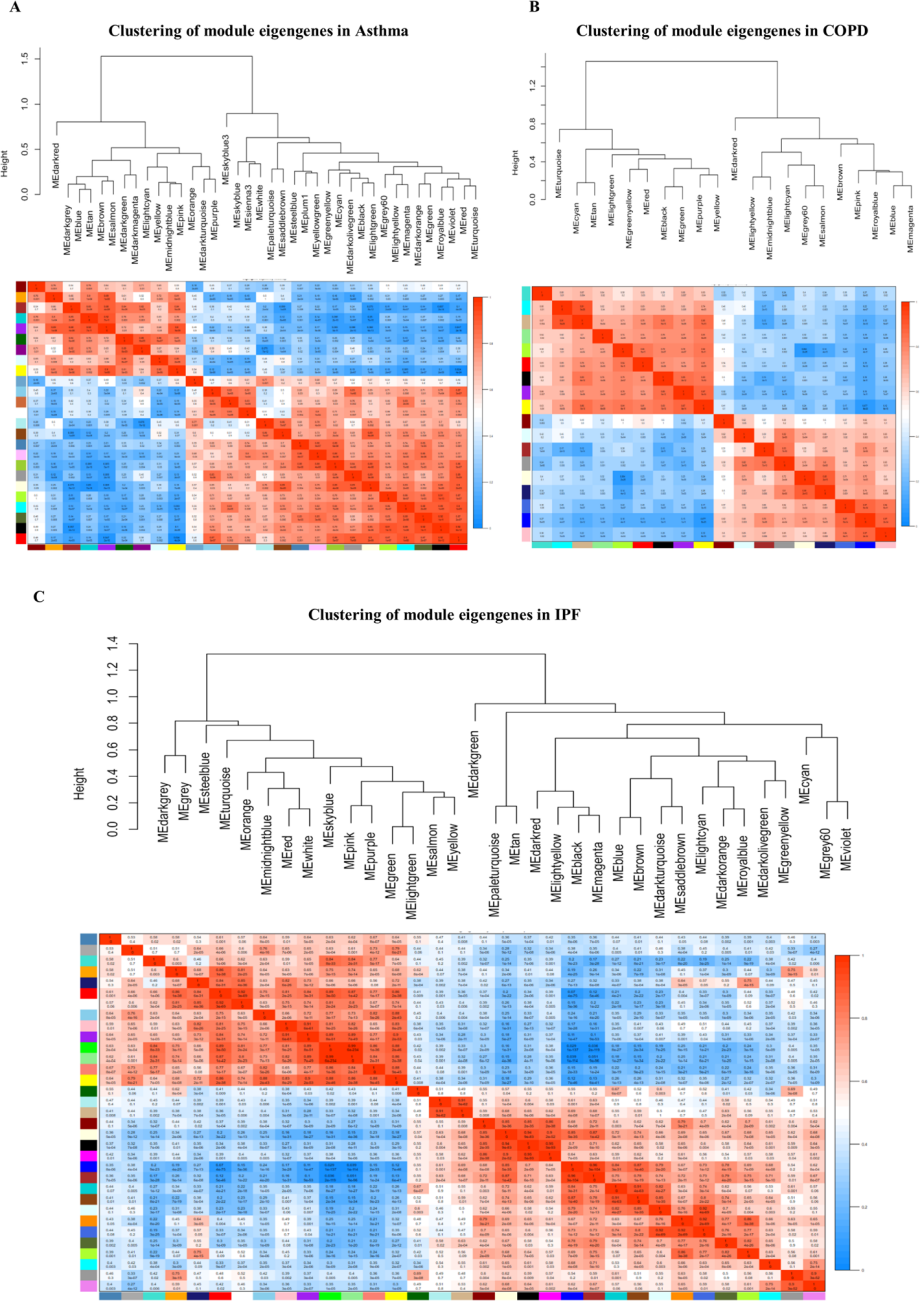
Clustering and heatmap module eigengenes. **a** Clustering and heatmap module eigengenes in asthma, **b** Clustering and heatmap module eigengenes in COPD, and **c** Clustering and heatmap module eigengenes in IPF

**Fig. 5:**
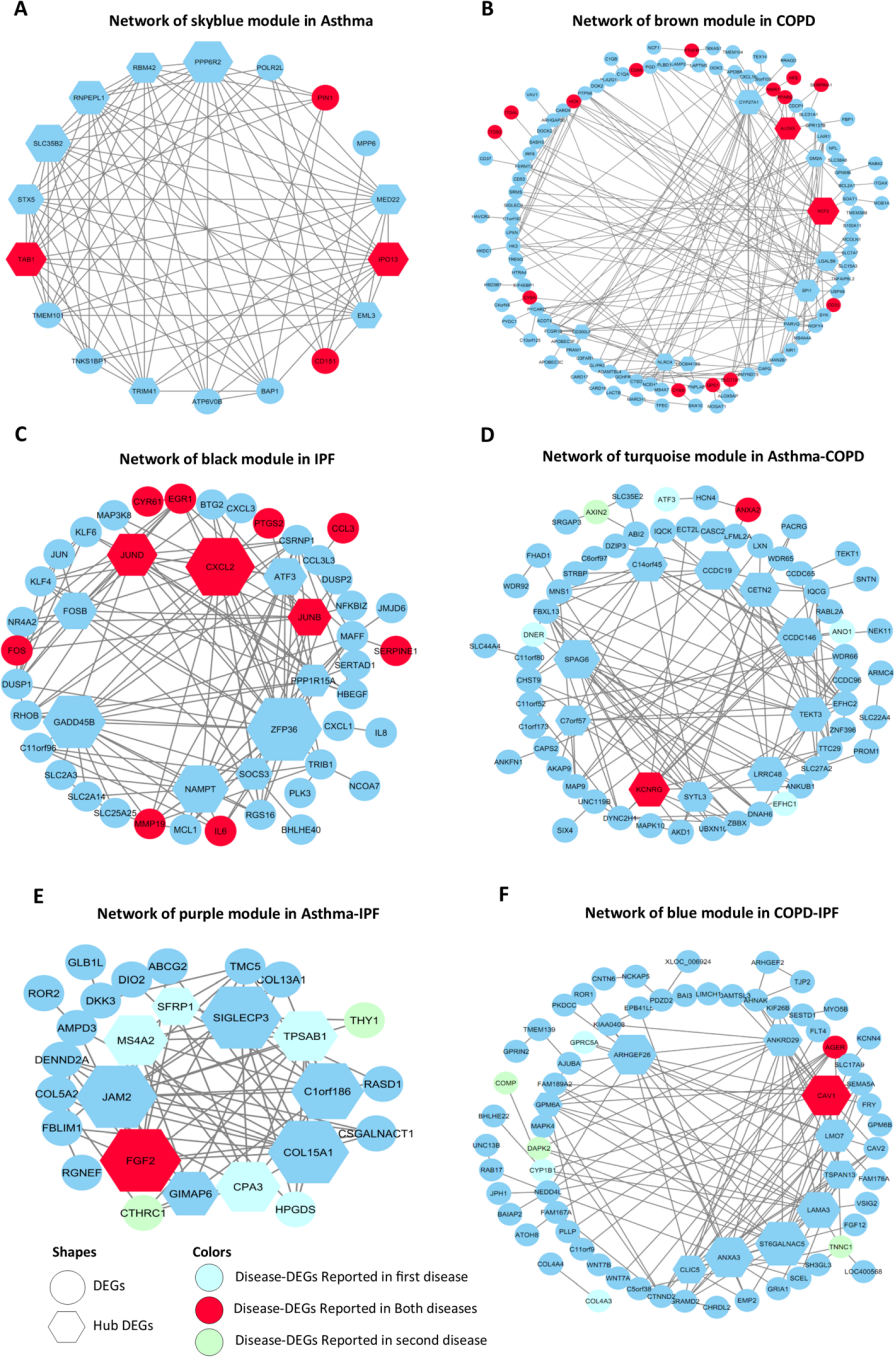
The network of the most important candidate modules among three diseases, in which the nodes represent DEGs. Hexagons represent the top 10 hubs of the module. Reported DEGs in DisGeNET are indicated in red. Novel DEGs are indicated in blue. The size of nodes reveals the degree level so that the higher degree is depicted by the larger size. **a** The network of the Skyblue module in asthma. In this module, 10 hub DEGs and 8 adjacency DEGs are involved. **b** The network of the brown module in COPD. In this module, 10 hub DEGs and 104 adjacency DEGs are implicated. **c** The network of the black module in IPF. In this module, 10 hub DEGs and 37 adjacency DEGs are involved. **d** The network of the turquoise module in the asthma-COPD in which red circles represent shared DEGs in asthma and COPD and, turquoise circles represent just DEGs in asthma and, green circles represent just DEGs in COPD. In this module, 10 hub DEGs and 57 adjacency DEGs are implicated. **e** The network of the turquoise module in the asthma-IPF in which red circles represent shared DEGs in asthma and IPF and, turquoise circles represent just DEGs in asthma and, green circles represent just DEGs in IPF. In this module, 10 hub DEGs and 17 adjacency DEGs are involved. **f** The network of the turquoise module in COPD-IPF in which red circles represent shared DEGs in COPD and IPF and, turquoise circles represent just DEGs in COPD and, green circles represent just DEGs in IPF. In this module, 10 hub DEGs and 61 adjacency DEGs are involved

Disease-specific DEGs (i.e., 1907, 1770, and 1832 DEGs for COPD, IPF, and asthma, respectively) were used to construct three disease-specific co-expression networks. The optimal β parameter for obtaining a scale-free network was computed as 11, 15, and 21 for COPD, IPF, and asthma, respectively (Additional file 2). Other parameters were mentioned in Section 2.3. Each disease-specific network was clustered in various modules, which are specified with unique colors (Fig. 3 a, b, and c). The DEGs not clustered in any module were color-coded as gray and eliminated from the networks. Then, The MEs representing the gene expression profile in the module were computed for each module in the three constructed disease-specific networks (Fig. 4). Thus, a total of 18, 25, and 33 modules were obtained for COPD, asthma, and IPF, respectively (Additional file 3). Then, three sequential steps including module-trait association, MM-GS, and disease-associated gene analyses were used to identify the most important modules for each disease (Section 2.3). One most important module as well as two and four modules were identified in asthma (skyblue), IPF (black, yellow), and COPD (black, brown, grey60, and midnightblue), respectively (Table 3). More detailed results are provided in Additional file 4. Next, the top 10 hub DEGs were identified in disease-specific candidate modules among the three diseases and the DEGs reported in DisGeNET and novel disease-DEGs were represented in the candidate modules (Fig. 5a, b, and c).

**Table 3:** The Final Result of MM-GS and Disease-associated Genes Analyses for Identifying the Most Important Modules in COPD, IPF, and Asthma after Second and Third Filters

Disease	Module	No. Genes	MM-GS		Disease-associated Genes	
			Correlation	*P*-value	Count	*P*-value
asthma	Skyblue	18	−0.66	0.0029	4	0.01
	Stellblue	16	0.5	0.049	2	0.37
	Darkgreen	29	0.44	0.017	0	0.17
	Lightyellow	101	0.26	0.0086	11	0.11
IPF	Blue	593	0.86	8*E* − 175	24	0.10
	Lightgreen	25	0.81	9.3*E* − 07	3	0.23
	Yellow	329	0.89	1.5*E* − 113	38	6*E* − 04
	Purple	51	0.87	1.2*E* − 16	5	0.28
	Red	129	0.85	3.7*E* − 37	4	0.20
	brown	508	0.88	1*E* − 165	24	0.34
	Salmon	37	0.61	6.1*E* − 05	3	0.59
	Green	202	0.84	5.2*E* − 55	11	0.81
	Black	121	0.68	9.7*E* − 18	19	1*E* − 04
	Pink	98	0.76	1.1*E* − 19	3	0.26
	lightyellow	25	0.87	1.6*E* − 08	1	0.70
COPD	Blue	678	0.85	2.4*E* − 190	47	0.08
	Pink	194	0.63	7.5*E* − 23	8	0.59
	Red	218	0.28	2.7*E* − 05	7	0.25
	Purple	125	0.67	1.3*E* − 17	7	0.35
	Turquoise	949	0.37	3.7*E* − 32	36	0.15
	Tan	78	0.63	6.4*E* − 10	7	0.15
	Brown	397	0.53	3.9*E* − 30	47	1*E* − 04
	Yellow	252	0.52	7.4*E* − 19	12	0.85
	Black	451	0.39	7.8*E* − 18	23	3*E* − 03
	Cyan	45	0.4	0.0065	4	0.27
	Grey60	83	0.43	5*E* − 05	10	0.01
	Green yellow	81	0.22	0.048	1	0.13
	Midnight blue	33	0.38	0.029	5	0.01
	Royal blue	12	0.58	0.048	0	0.45

### Consensus network analysis (CNA)

Three Consensus Networks (CNs) were constructed, including asthma-COPD, COPD-IPF, and asthma-IPF. Each CN was used to extract the Consensus Modules (CMs). The plots of β parameter computation for the constructed CNs are provided in Additional file 5. A unique color was assigned to each CM (Fig. 3d, e, and f) and CMs with an eigengene correlation value of ≥0.85 were combined as well. Similarly, the number of CMs was 7, 2, and 4 for asthma-COPD, asthma-IPF, and COPD-IPF, respectively. The details of the CMs are presented in Additional file 6. Then, the Module-Trait associations for CMs were calculated, the final results of which are summarized in Table 4. Afterward, the top 10 hub genes were identified in the candidate CMs. Finally, the reported DEGs in DisGeNET and novel disease-DEGs were illustrated in the candidate CMs (Fig. 5d, e, and f).

**Table 4:** Module-trait Association Analysis Modules, Consensus Modules Derived from COPD, IPF, and asthma

Consensus	Module	Correlation	*P*-value	Number of Genes
asthma-COPD	Pink	0.62	1*E* − 04	35
	Purple	−0.5	0.004	27
	Turquoise	−0.5	0.003	124
	Blue	−0.53	3*E* − 04	97
asthma-IPF	Yellow	0.52	0.003	57
	Purple	0.57	7*E* − 04	37
COPD-IPF	Blue	−0.67	2*E* − 10	103
	Brown	−0.52	4*E* − 06	94
	Green	0.55	1*E* − 06	38

A CN including 304 shared DEGs was reconstructed to extract the CMs among the three diseases. However, no CM could be extracted using the WGCNA.

### Enrichment analysis

Enrichment analysis was performed to functionally evaluate the gene sets that were involved in specific modules, CMs, and overlapping DEGs among three diseases as follows.

#### Enrichment analysis of disease-specific modules

Table 5 presents some of the significant biological pathways and GO biological processes that belong to the modules obtained in each disease, the details of which are reported in Additional file 7.

**Table 5:** Results of the Pathway and GO Biological Process Enrichment Analysis for Several Important Modules

Disease	Module	Pathway/BP	Term	Adjusted *P*-value
IPF	Black	Pathway	TNF signaling pathway	2.01*E* − 014
			Cytokine Signaling in Immune system	1.31*E* − 05
			Salmonella infection	4.49*E* − 09
			Legionellosis	1.81*E* − 04
			Inflammatory bowel disease	3.39*E* − 05
			p53 signaling pathway	4.69*E* − 03
			Apoptosis	7.32*E* − 03
			Epithelial cell signaling in Helicobacter pylori infection	2.86*E* − 02
		BP	chronic inflammatory response	2.83*E* − 07
			inflammatory response to wounding	2.83*E* − 07
			cellular response to cytokine stimulus	1.57*E* − 06
			response to interleukin-1	1.74*E* − 05
			positive regulation of cell death	2.26*E* − 05
			fat cell differentiation	1.35*E* − 04
asthma	Skyblue	Pathway	TGF beta signaling pathway	3.19*E* − 02
			NF-kB Signaling Pathway	3.19*E* − 02
			WNT Signaling Pathway	3.19*E* − 02
			Toll-Like Receptor Pathway	3.19*E* − 02
			Signal transduction through IL1R	3.19*E* − 02
		BP	No Significant	–
COPD	Brown	Pathway	Tuberculosis	6.85*E* − 09
			*Staphylococcus aureus* infection	5.2*E* − 05
			Leishmaniasis	8.33*E* − 06
			Pertussis	5.3*E* − 04
			Legionellosis	4.63*E* − 03
			ROS, RNS production in response to bacteria	3.61*E* − 04
			Cell surface interactions at the vascular wall	4.26*E* − 04
			Innate immune system	4.33*E* − 03
		BP	Neutrophil degranulation	3.84*E* − 023
			Regulation of inflammatory response to antigenic stimulus	5.28*E* − 05
			Regulation of innate immune response	9.80*E* − 05
			positive regulation of interleukin-6 secretion	2.52*E* − 04
			Chronic inflammatory response	7.24*E* − 03
			Inflammatory response to wounding	7.24*E* − 03
			Positive regulation of tumor necrosis factor secretion	5.28*E* − 05

#### Enrichment analysis of CMs

Some of the enriched significant biological pathways and GO biological processes of the CMs are reported in Table 6, and related details are provided in Additional file 8.

**Table 6:** Results of the Pathway and GO Biological Process Enrichment Analysis of Several Important Consensus Modules

Consensus	Module	Pathway/BP	Term	Adjusted *P*-value
asthma-IPF	Purple	Pathway	Extracellular matrix organization	6.81*E* − 06
			Degradation of the extracellular matrix	2.84*E* − 02
			Collagen biosynthesis and modifying enzymes	1.48*E* − 02
			Integrin cell surface interactions	2.57*E* − 04
			Collagen formation	2.14*E* − 02
			Syndecan interactions	2.14*E* − 02
			Non-integrin membrane-ECM interactions	5.91*E* − 02
		BP	extracellular matrix disassembly	2.47*E* − 05
			biofilm matrix organization	2.47*E* − 05
			basement membrane organization	2.47*E* − 05
			negative regulation of wound healing, spreading of epidermal cells	3.49*E* − 02
			extracellular matrix organization	2.47*E* − 05
			regulation of blood vessel branching	5.56*E* − 02
asthma-COPD	Turquoise	Pathway	Assembly of the primary cilium	4.21*E* − 02
		BP	cilium assembly	1.30*E* − 04
			cilium organization	1.30*E* − 04
			plasma membrane bounded cell projection assembly	6.81*E* − 04
			cilium-dependent cell motility	2.37*E* − 03
			regulation of cilium movement	2.37*E* − 03
COPD-IPF	Blue	Pathway	Focal adhesion	7.89*E* − 05
			Regulators of Bone Mineralization	2.33*E* − 04
			VEGF, Hypoxia, and Angiogenesis	2.67*E* − 03
			ECM-receptor interaction	1.7*E* − 03
			Actions of Nitric Oxide in the heart	4.82*E* − 03
			Cell junction organization	5.75*E* − 02
			Basal cell carcinoma	2.03*E* − 02
			Bacterial invasion of epithelial cells	4.96*E* − 02
			Small cell lung cancer	5.97*E* − 02
			Integrin cell surface interactions	5.42*E* − 02
			VEGF binds to VEGFR leading to receptor dimerization	5.42*E* − 02
			VEGF ligand-receptor interactions	5.42*E* − 02
			Cell junction organization	5.75*E* − 02
		BP	sprouting angiogenesis	2.66*E* − 02
			activation of Janus kinase activity	2.34*E* − 02
			extracellular matrix assembly	2.66*E* − 02
			endothelial stalk cell fate specification	2.66*E* − 02
			lung vasculature development	3.60*E* − 02
			positive regulation of focal adhesion assembly	2.48*E* − 02
			positive regulation of endothelial cell chemotaxis	7.16*E* − 03
			collagen fibril organization	2.66*E* − 02
			positive regulation of endothelial cell proliferation	4.81*E* − 02

#### Enrichment analysis of overlapping DEGs among three diseases

**Fig. 6:**
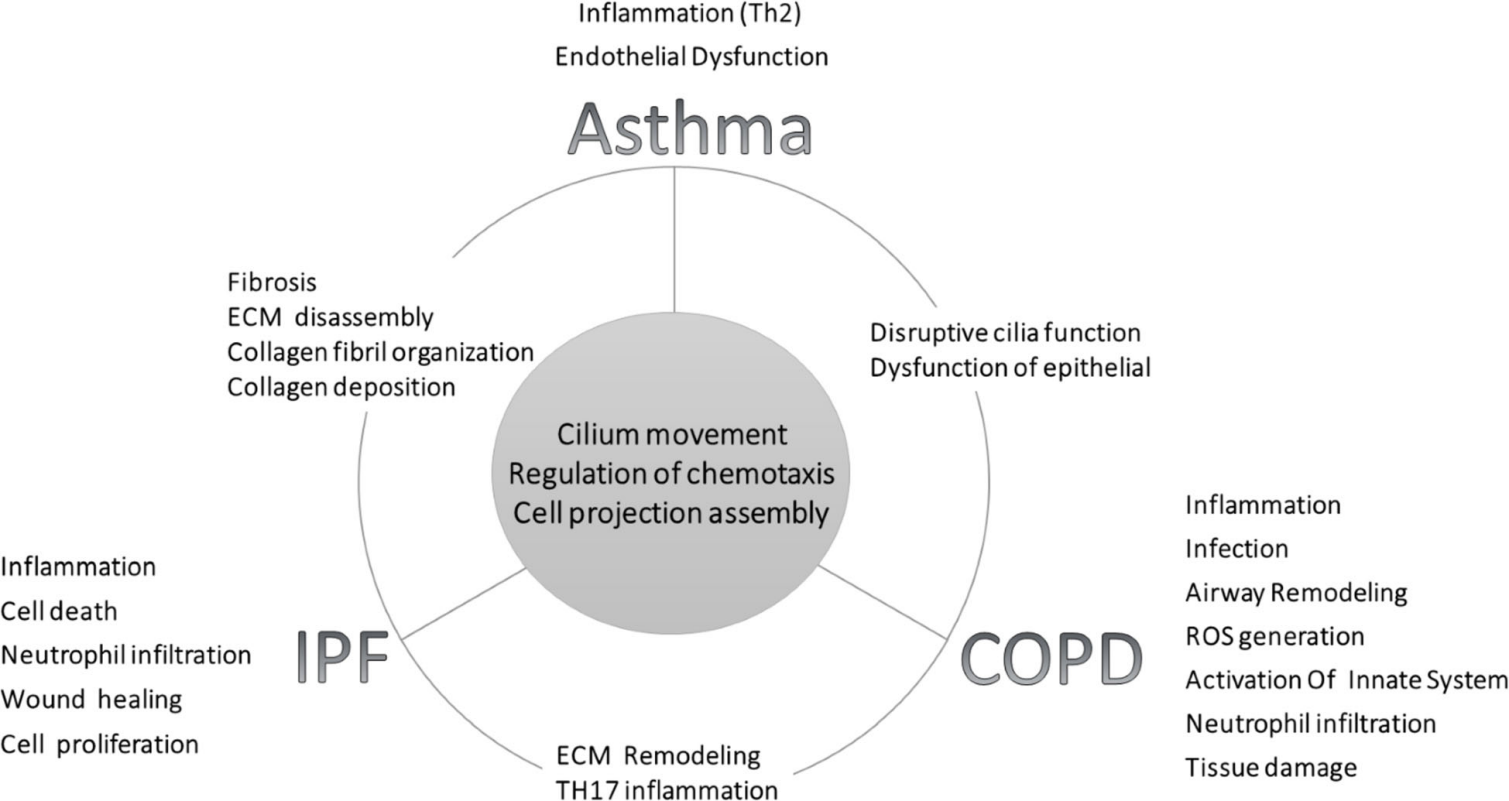
Summary of functional enrichment of candidate disease-specific and consensus modules among three diseases

Table 7 summarizes some of the enriched significant biological pathways and GO biological processes of the overlapping DEGs among three diseases and the detailed results are reported in Additional file 9. Figure 6 is overall representation of functional enrichment of candidate disease-specific, CMs, and overlapping DEGs among three diseases.

**Table 7:** Results of the Pathway and GO Biological Process Enrichment Analysis of Overlapping DEGs among Three Diseases

Pathway/BP	Term	Adjusted *P*-value
Pathway	No Significant	–
BP	Cilium movement	7.18*E* − 05
	Plasma membrane bounded cell projection assembly	1.39*E* − 02
	Positive regulation of positive chemotaxis	3.42*E* − 02
	Flagellated sperm motility	4.31*E* − 02
	Cilium organization	4.31*E* − 02
	Regulation of positive chemotaxis	4.39*E* − 02

### Identification of candidate drug-target networks

#### Identification of diseases-specific candidate drug-target networks

**Fig. 7:**
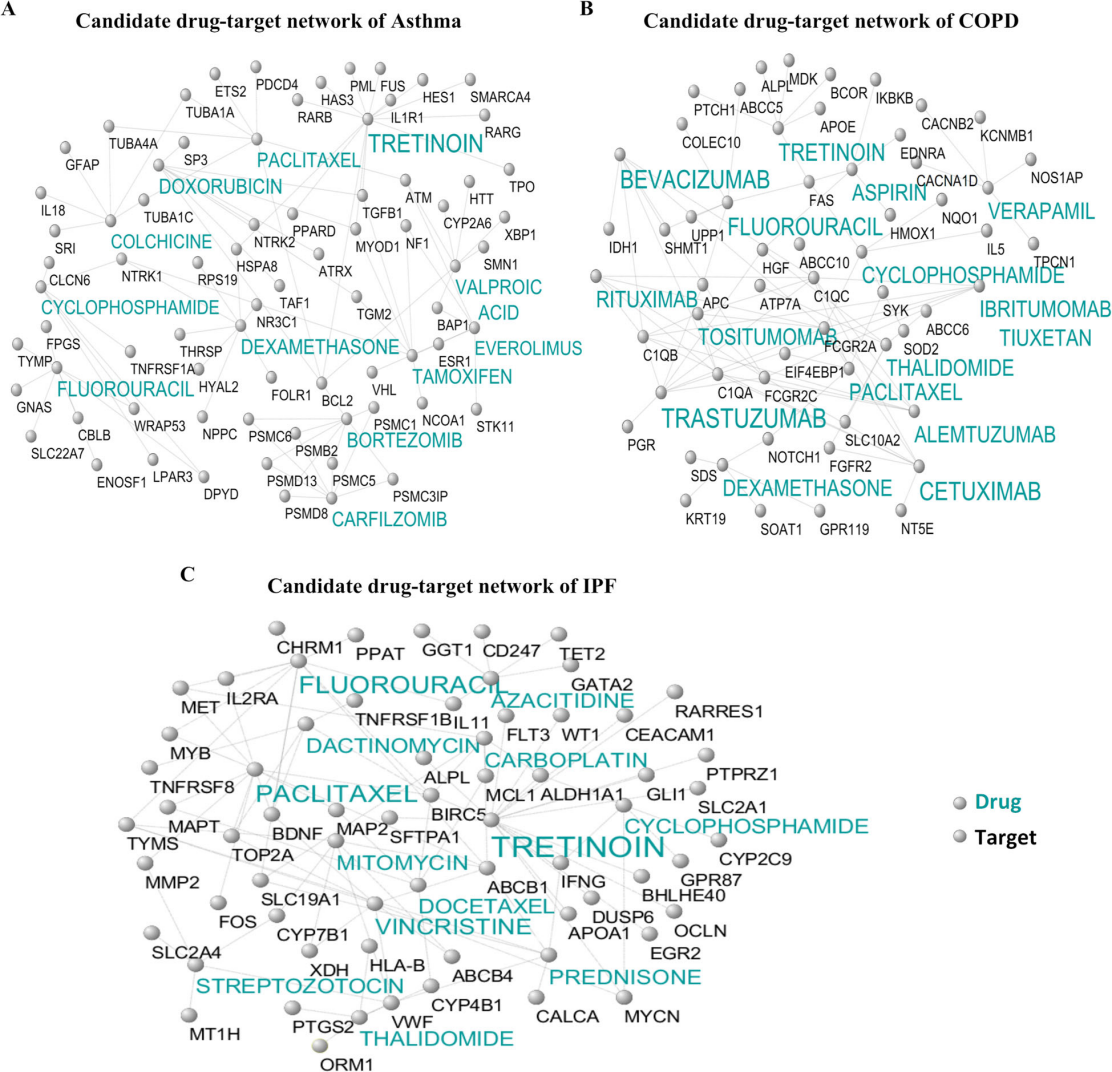
Candidate disease-specific drug-target networks. Label targets and drugs are colored with black and turquoise, respectively. The size of drug letters reveals the degree of drug. **a** Candidate drug-target network of asthma, **b** Candidate drug-target network of COPD, and **c** Candidate drug-target network of IPF

The candidate drug-target interactions were investigated after identifying consensus and specific modules among three diseases. Figure 7a, b, and c illustrates the candidate drug-target networks that were constructed based on specific DEGs (three diseases) from the DGIdb database. The number of interactions, the number of genes, and the number of targets in asthma, COPD, and IPF candidate drug-target networks were 335, 285, and 192, as well as 114,100, and 83, and finally, 150, 137, and 60, respectively. The low-level interactions were removed to simplify the illustration. The detailed results of drug-target interactions in Fig. 7a, b, and c are reported in Additional file 10.

#### Identification of candidate consensus drug-target networks

**Fig. 8:**
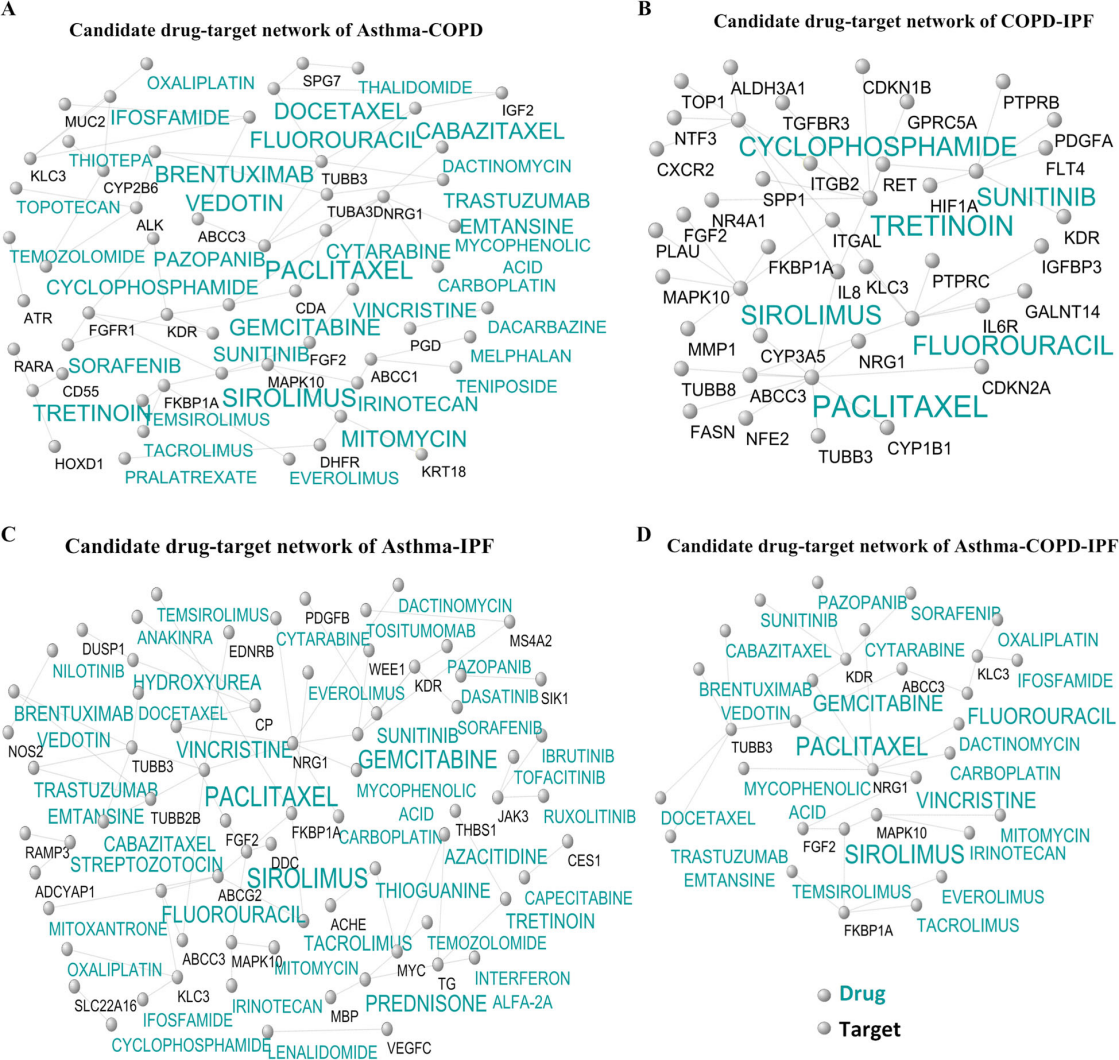
Candidate shared drug-target networks. Label targets and drugs are colored with black and turquoise, respectively. The size of drug letters represents the degree of drug. **a** Candidate drug-target network of asthma-COPD, **b** Candidate drug-target network of asthma-IPF, **c** Candidate drug-target network of COPD-IPF, and **d** Candidate drug-target network of the asthma-COPD-IPF

Figure 8a, b, and c displays the candidate consensus drug-target networks constructed based on common DEGs between two diseases. The number of interactions was 62, 69, and 134 in the asthma-COPD, asthma-IPF, and COPD-IPF candidate consensus drug-target networks. Furthermore, the number of genes and the number of targets were 24, 30, and 63, as well as 33, 42, and 53 in asthma-COPD, asthma-IPF, and COPD-IPF candidate consensus drug-target networks, respectively. The low-level interactions were removed to simplify the illustration as well. The detailed results of drug-target interactions are reported in Additional file 11.

#### Identification of candidate shared drug-target networks

Figure 8 d depicts the candidate shared drug-target network constructed based on common DEGs among three diseases (Additional file 12). The numbers of, genes, targets and their interactions in this network were 8, 23, and 30, respectively.

## Discussion

Progressive chronic respiratory diseases are associated with a persistent and progressive decline in lung function and exacerbations, which require smart diagnosis and treatment. Decreasing the rate of progression, staging, and exacerbations in chronic respiratory diseases such as asthma, COPD, and IPF is critically vital in controlling mortality (Murray et al. 2017). The present paper employed a systems biology approach to determine specific and shared biological biomarkers of COPD, asthma, and IPF, along with the candidate drugs, followed by analyzing the lung transcriptomic similarity among three diseases. The disease-specific, consensus, and shared DEGs among the three diseases were extracted after transcriptomic data analysis. The identified DEGs in each comparison were used for constructing co-expression networks and identifying the functional modules. Based on the results, one module (Skyblue) in asthma, two modules in IPF (black and yellow), and four modules in COPD (brown, gray60, black, and midnightblue) were identified.

The analysis of biological pathways suggested that the genes of the Skyblue module in the asthma-specific are significantly associated with NF-kB, Wnt, Toll-like receptor, and transforming growth factor (TGF)-beta signaling pathways. These pathways are involved in the inflammation of the respiratory tract, immune system, and chronic inflammatory diseases (Li et al. 2018; Zhang et al. 2018; Dai et al. 2018). In the Skyblue module, eight hub DEGs including STX5, SLC35B2, RNPEPL1, RBM42, PPP6R2, MED22, EML3, and TRIM41 are novel for asthma. In addition, RBM42 in this module is the component of stress granules (Han et al. 2010) which appear when the cell is under the stress. STX5 is another hub DEG that exists in the nicotine pathway. TRIM41 is another proposed hub DEG, which is an intrinsic immune factor that inhibits influenza A virus infection and is expressed in lung epithelial cells (Patil et al. 2018). According to the results, RBM42, STX5, and TRIM41 may have critical functions in asthma.

The biological pathways enriched for the COPD-specific module genes suggest inflammatory pathways, infections, and remodeling pathways. For example, the genes of the brown module are significantly associated with the biological pathways of Tuberculosis, *Staphylococcus aureus* infection, Leishmaniasis, Pertussis, and Legionellosis. All these pathways play critical roles in infections and remodeling in COPD (Carette et al. 2018; Tsenova et al. 2014; Ziesemer et al. 2018; Sabulski et al. 2017). In the brown module, eight hub DEGs including CYp27A1, GM2A, LGAL59, SPI1, PARVG, LOC644189, NLRC4, CD300LF are considered as the novel genes in the COPD. Furthermore, the CYP27A1 in this module is an initiating enzyme in the acidic pathway to bile acids (Beck et al. 2019). In macrophages, 27-hydroxycholesterol is generated by this enzyme and may be helpful against the production of inflammatory factors associated with cardiovascular diseases (Taylor et al. 2010). Moreover, GM2A is a lipid transfer protein that stimulates the enzymatic processing of gangliosides and activates T-cell through lipid presentation. Additionally, it significantly correlates with alcohol dependence and nicotine dependence (Xiang et al. 2019). Similarly, LGALS9 encodes human galectin-9, which is expressed in various tumor cells. The expression of TNF-α, IL-1β, and IL-6 increases significantly in monocytes that are stimulated with Galectin-9 (Wang et al. 2019a). Moreover, the interaction of Galectin-9 with CD40 on T-cells induces their proliferation inhibition and cell death (Vaitaitis and Wagner Jr 2012). Likewise, SPI1 is a transcription factor that is involved in the development of several different types of immune lineage precursor cells, including T-cells, B-cells, dendritic cells (DCs), and monocytes (Merad et al. 2013; Yashiro et al. 2019). In addition, SPI1 knockdown decreases the expression of C-C chemokine receptor type 7 (CCR7) which is crucial for the migration of DCs to draining lymph nodes (Yashiro et al. 2019). Furthermore, NLRC4 is involved in the formation of inflammasome and is also crucial for the production and secretion of IL-1β. Therefore, the production of the inflammatory cytokine IL-1β increases in tissue damage, environmental stress, infection, or chronic inflammation (Wang et al. 2019b). Thus, CYP27A1, GM2A, LGALS9, SPI1, and NLRC4 may provide a novel therapeutic strategy and are suggested as the candidates in COPD.

The results of the biological process analysis for IPF-specific genes demonstrate that the genes of the black module are enriched in chronic inflammatory responses, as well as cell proliferation, cell death, neutrophil processes, and wound healing. In this module, seven genes including FOSB, ATF3, PPP1R5A, ZFP36, SOCS3, NAMPT, and GADD45B are novel in IPF., ATF3 is a candidate hub DEG that changes the expression of a subset of inflammatory genes downstream of IFN signaling and has a prominent role in macrophages. Furthermore, cytokines and IFNs are considered as the downstream of innate immune pathways and play a critical role in the immune response to microbial infection, leading to chronic inflammatory conditions and autoimmune diseases (Labzin et al. 2015). The PPP1R15A is another hub DEG which encodes the human growth arrest and DNA damage-inducible protein (GADD34). Moreover, the overexpression of GADD34 facilitates apoptosis in mammalian cells following DNA damage and other cell stresses (Brush et al. 2003). Additionally, the overexpression of GADD34 and ATF4 induces CHOP expression, inhibits *Bcl-2* expression, and causes damage to oxygen free radicals, inducing apoptosis and endoplasmic reticulum stress (Wang et al. 2019c). Similarly, ZFP36 is another identified hub DEG which is associated with innate immunity, including type I interferon signaling pathway, viral response, and inflammation (Mahmoud et al. 2019). Moreover, ZFP36 regulates the positive regulation of the I-κB/NF-κB cascade and the TRIF-dependent toll-like receptor, as well as MAPK, TNF, and T-cell receptor signaling pathways (Tu et al. 2019). SOCS3 in the black module is known as the suppressor of cytokine signaling and is a master negative regulator of toll-like receptor 4 as well (Bae et al. 2019). Furthermore, TGF-β2 could strongly inhibit IL-6-induced STAT3 activation and synergy with IL-6, resulting in enhanced SOCS3 expression (Du et al. 2019). In addition, NAMPT promotes B-cell maturation while inhibiting neutrophil apoptosis (Revollo et al. 2007). Likewise, GADD45B is induced by genotoxic stress and implicated in genotoxic stress-induced responses, cell cycle arrest, inducing apoptosis, and DNA damage repair (Gupta et al. 2006). Therefore, genes like ATF3, PPP1R15A, ZFP36, SOCS3, NAMPT, and GADD45B may provide a novel therapeutic strategy for IPF.

The consensus modules between all disease pairs (i.e., asthma-COPD, asthma-IPD, and COPD-IPF) were investigated after evaluating the disease-specific modules. Also, two modules (Turquoise and Pink) for asthma-COPD, one module (Purple) for asthma-IPF, and three modules (Brown, Green, and Blue) for COPD-IPF pairs were identified after the module-trait analysis. Biological pathways and GO analysis conducted for the genes, are involved in each significant consensus module.

Based on the results of the biological process analysis for the asthma-COPD, the genes of the turquoise module were mainly enriched in cilium assembly, cilium organization, cilium movement, and plasma membrane-bounded cell projection assembly. These processes were due to the dysfunction of the bronchial epithelial and the disruption of cilia function as the important features of asthma and COPD diseases (Wan et al. 2016; Yaghi and Dolovich 2016). Among ten hub DEGs identified for the asthma-COPD, nine DEGs including SPAG6, C7orf57, SYTL3, LRRC48, TEKT3, CCDC146, CETN2, CCDC19, and C14orf45 were identified as the novel genes. The phenotypic abnormalities and mutations in LRRC48 cause defects in the motile cilia function (Ha et al. 2016). Furthermore, the lack of CETN2 leads to olfactory cilia loss and the impaired ciliary trafficking of olfactory signaling proteins (Ying et al. 2014). Therefore, LRRC48 and CETN2 can be considered as candidate predictive biomarkers for the asthma-COPD.

The results of the biological process analysis for asthma-IPF showed that the genes of the purple module were enriched in extracellular matrix disassembly, biofilm matrix organization, cellulose microfibril organization, and collagen fibril organization. Five novel hub DEGs (i.e., JAM2, SIGLECP3, C14orf186, COL15A1, and GIMAP6) were identified in asthma-IPF among ten hub DEGs. In the purple module, COL15A1 is involved in the smooth muscle cell (SMC) phenotype that is controlled by the epigenetic state, so that the hypomethylation of COL15A1 transpires during SMC proliferation and the consequent increased gene expression may affect the SMC phenotype (Connelly et al. 2013). Moreover, GIMAP6 is another proposed DEG in this module, which is strongly expressed in the adaptive immune system and T-cell lines. Similarly, it plays a role in changing the immune function by controlling cell death and T-cell activation (Ho and Tsai 2017). Additionally, JAM2 acts as an adhesive ligand for interacting with a variety of immune cell types. Likewise, it is a member of junctional adhesion molecules (JAMs). JAMs, located at the tight junctions of epithelial and endothelial cells, are variably expressed by SMCs, platelets, erythrocytes, and leukocytes in humans. In addition, JAMs are involved in the regulation of diverse functions like cell permeability, polarity, and proliferation, metastasis, or leukocyte transmigration during tissue inflammation (Hintermann et al. 2018). Accordingly, COL15A1, GIMAP6, and JAM2 may present a novel therapeutic strategy for the asthma-IPF.

The results of the biological process analysis for COPD-IPF represented that the genes of the blue module are enriched in immune processes, inflammation, and remodeling. Nine novel hub DEGs were specified in COPD-IPF, including GPM6A, ARHGEF26, ANKRD29, LMO7, TSPAN13, LAMA3, ST6GALNAC5, ANXA3, and CLIC5. In the blue module, LMO7 is known as a negative feedback regulator of TGF-β signaling and extracellular matrix deposition (Xie et al. 2019). Moreover, the cytokine TGF-β has a critical role in remodeling, wound healing, and fibrotic diseases (Xie et al. 2019). Also, TSPAN13 in this module belongs to the Tetraspanin family, which is involved in a variety of biological processes including differentiation, cell development, activation, growth factor, motility, proliferation, and metastasis (Wang et al. 2019d). LAMA3 is another proposed hub DEG which is induced by hypoxia in macrophages (Gang et al. 2017). Moreover, recessive mutations in the LAMA3 that encode laminin332 (LM332) affect epithelial adhesion (Condorelli et al. 2018). Additionally, ANXA3 is a part of the Annexin family and binds to the phospholipids in a *Ca*^2+^-dependent manner. It also plays a vital role as a mediator in cellular growth, signal transduction pathways, cancer proliferation, invasion, and migration (Wang et al. 2019e). Therefore, LMO7, TSPAN13, LAMA3, and ANXA3 are suggested as the candidates in COPD-IPF.

Previous studies have only evaluated the overlap mechanism between two diseases whereas the present study considered three diseases together and could identify the focal biological processes for all three diseases. These results were not observed due to the presence of other genes available in the top rank between two diseases, and the statistical analysis led to these processes that were not shared in the overlap of two diseases. Likewise, the processes observed in COPD and IPF confirmed the irreversibility of these diseases whereas a cure is suggested in asthma.

Based on the data in Tables 2, 230 shared DIPs were found in the asthma-COPD. This may be due to the presence of inflammation, airway remodeling, and obstruction in both diseases (Soriano et al. 2017; Postma and Rabe 2015). One-hundred seven shared DIPs presented in COPD-IPF may suggest less transcriptional similarity between these two diseases compared to asthma-COPD. Nevertheless, overlapping genes and pathways exist between two diseases due to inflammation and the reduction of oxygenation in different tissues (O’Donnell et al. 2016). Furthermore, the presence of 85 shared DIPs in asthma-IPF indicates the least transcriptional similarity between these two diseases due to the presence of inflammation and shared inflammatory pathways in both diseases (Soriano et al. 2017; Vestbo et al. 2013).

Drug-target identification for consensus and common DEGs in three diseases revealed that based on specific DEGs, tretinoin as a retinoid drug is recommended for all three diseases. Retinoids are involved in many essential functions of the body such as the immune system, cell proliferation, and differentiation, bone growth, the activation of tumor suppressor genes, as well as natural and malignant epithelial cell growth in various tissues (Buckley et al. 2006; Schultze et al. 2014). Moreover, they may be useful in the treatment of the studied lung diseases due to their anticoagulant, antimicrobial, and anti-inflammatory effects, along with their impact on the immune system (Buckley et al. 2006). According to ref. (Jiang et al. 2017), dexamethasone is a candidate drug for COPD and asthma and is considered as the glucocorticoid that is among the most effective drugs for controlling airway inflammation. Additionally, this drug inhibits the inflammation of asthma and COPD by suppressing the cytokine expression (i.e., IL-4 and IL-13). Tamoxifen is considered as another drug candidate for asthma that can improve this disease by preventing cell growth, inducing apoptosis in neoplastic cells, and reducing inflammation in the lung (Perez et al. 2016).

Bevacizumab is one of the VEGF inhibitors that can be identified as a candidate for COPD (Papaioannou et al. 2006). Similarly, verapamil is another drug candidate for COPD and is a calcium channel blocker which attaches to the alpha-subunits of L-type calcium channels and thus inhibits the pathway of calmodulin and CaM kinase from the mucin genes. In addition, it is a useful drug for reducing mucus production while improving bronchial inflammation (Khakzad et al. 2012).

Low-dose paclitaxel is one of the TGF-β1/Smad3 pathways suppressing drugs, and in this study, it was identified as a candidate drug for IPF. TGF-β1 is responsible for creating a cascade of incidents leading to fibrosis, and the abnormal activation of TGF-β1 plays a vital role in the pathogenesis of IPF (Wang et al. 2013). Therefore, the use of paclitaxel can be suggested as one of the most effective medications for this disease. Prednisone is another useful drug in the treatment of IPF. It is predicted as a candidate in this study as well. This drug increases caveolin-1 while decreasing TNF-α, TGF-β1, and PDGF (Yu et al. 2017). Furthermore, it has anti-inflammatory properties and suppressive effects on the immune system and thus is used to treat IPF (Li et al. 2017).

Likewise, sirolimus as an immunosuppressant drug is suggested as a candidate for the asthma-COPD therapy. This drug inhibits the activation of T and B-cells by inhibiting mTOR and plays an essential role in the biogenesis of the ribosome, cell cycle progression, lipid synthesis, mitochondrial biogenesis, and autophagy of COPD (Miłkowska-Dymanowska et al. 2017; Mukherjee and Mukherjee 2009). Moreover, it inhibits IL-5 cytokine, which plays an important role in regulating asthma severity (Powell et al. 2001). Therefore, sirolimus can be proposed as a shared drug in the treatment of COPD and asthma. Similarly, gemcitabine is another candidate drug in the asthma-COPD and its intranasal administration significantly reduces viruses and the inflammation of lungs and the anti-inflammatory cytokines including TNF-α and IL-1b. Therefore, it is effective in reducing the severity of asthma and COPD (Song et al. 2017).

Tacrolimus is one of the candidate medicines in asthma-IPF as well. This drug inhibits the transcription of IL-2 and several other cytokines, especially in T-helper lymphocytes (Horita et al. 2011). Additionally, combining this drug with methylprednisolone reduces the severity of the IPF (Horita et al. 2011). Tacrolimus also reduces inflammatory cells in the lung tissue and prevents asthma (Qiao et al. 2017). Sunitinib is considered as another candidate drug in asthma-IPF, which is known as one of the inhibitors of tyrosine kinases. Tyrosine kinases play an essential role in the pathogenesis of the IPF. Furthermore, this drug can block the process of Epithelial to Mesenchymal Transition (EMT) by TGF-β, and EMT plays a vital role in the development of IPF (Huang et al. 2016). Sunitinib also reduces mucus and inflammation and repairs remodeling in asthmatic patients (Huang et al. 2009). Therefore, it can be proposed as an effective treatment for asthma and IPF.

In addition, cyclophosphamide is regarded as one of the candidate drugs for COPD-IPF. The combination of this drug with corticosteroids is used to reduce the severity of IPF (Emura and Usuda 2016; Novelli et al. 2016). It should be noted that sunitinib, sirolimus, and tacrolimus drugs, can be applied as an effective treatment for asthma, COPD, and IPF due to the inhibitory effect of the tyrosine kinase and immune system.

The current study proposed unique and common biomarkers and drugs among the three diseases. Moreover, it was confirmed that asthma lung transcriptome is more similar to COPD than IPF. These findings could help the researchers to differentiate the mentioned diseases. However, this study has some limitations including ignoring the side effects of the drugs. Additionally, novel biomarkers were proposed computationally. Therefore, the clinical studies are recommended to confirm the validity and reliability of the results of this study, as well as those of similar studies in the future.

## Conclusions

The findings indicated that the proposed biomarkers in chronic lung diseases might serve as predictive targets among the three diseases. They include RBM42, STX5, and TRIM41 in asthma, CYP27A1, GM2A, LGALS9, SPI1, and NLRC4 in COPD, as well as ATF3, PPP1R15A, ZFP36, SOCS3, NAMPT, and GADD45B in IPF. In addition, other identified genes were LRRC48 and CETN2 in asthma-COPD, COL15A1, GIMAP6, and JAM2 in asthma-IPF, along with LMO7, TSPAN13, LAMA3, and ANXA3 in COPD-IPF. Finally, anti-inflammatory drugs such as corticosteroids were proposed for treating the three diseases.

## Supplementary information

**Additional file 1.** The list of DIPs and shared DEGs among three diseases.

**Additional file 2 **Selecting the optimal *β* parameter for disease-specific networks.

**Additional file 3.** The list of all gene symbols in candidate disease-specific modules.

**Additional file 4.** The list of selected modules for three diseases.

**Additional file 5 **Selecting the optimal *β* parameter for consensus networks.

**Additional file 6.** The list of all gene symbols in candidate consensus modules.

**Additional file 7.** Significant biological pathways and GO Biological Processes in disease-specific modules.

**Additional file 8.** Significant biological pathways and GO Biological Processes in consensus modules.

**Additional file 9.** Significant GO biological processes of overlapping DEGs among three diseases.

**Additional file 10:.** The list of candidate disease-specific drug-target interactions.

**Additional file 11.** The list of candidate consensus drug-target interactions.

**Additional file 12.** The list of candidates shared drug-target interactions among three diseases.

## Data Availability

The material is available at GEO# GSE47460 and GSE23611.

## Abbreviations

Bicor: Biweight midcorrelation
CM: Consensus module
CN: Consensus network
CNA: Consensus network analysis
COPD: Chronic obstructive pulmonary disease
DEG: Differentially expressed genes
DGIdb: Drug gene interaction database
DIP: DEG with identical pattern
GEO: Gene expression omnibus
GO: Gene ontology
GS: Gene significance
IPF: Idiopathic pulmonary fibrosis
KEGG: Kyoto encyclopedia of genes and genomes
ME: Module eigengene
MM: Module membership
TOM: Topological overlap matrix
WGCNA: Weighted gene co-expression network analysis
WHO: World Health Organization
