# Supplementary material for: Identification of biomarkers in common chronic lung diseases by co-expression networks and drug-target interactions analysis
Source: Mol Med. 2020 Jan 17;26:9. doi: 10.1186/s10020-019-0135-9 (PMC6969427; doi:10.1186/s10020-019-0135-9)

Selecting optimal β parameter for COPD

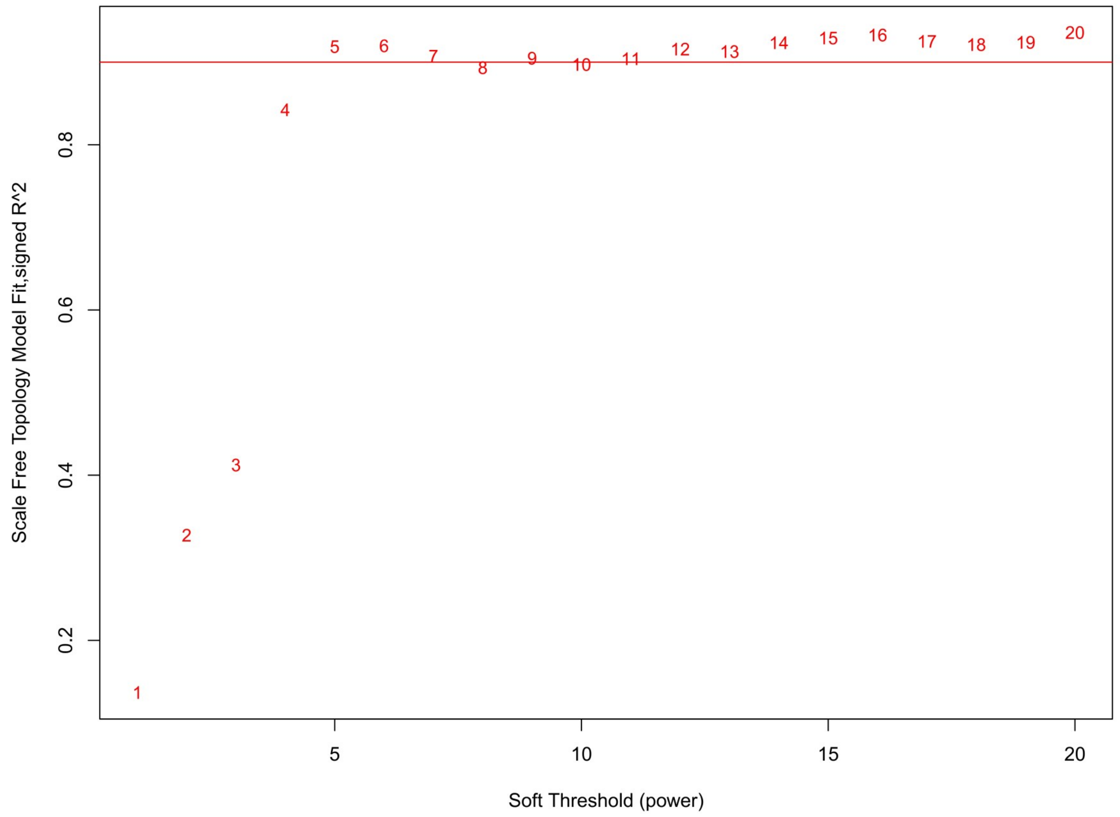

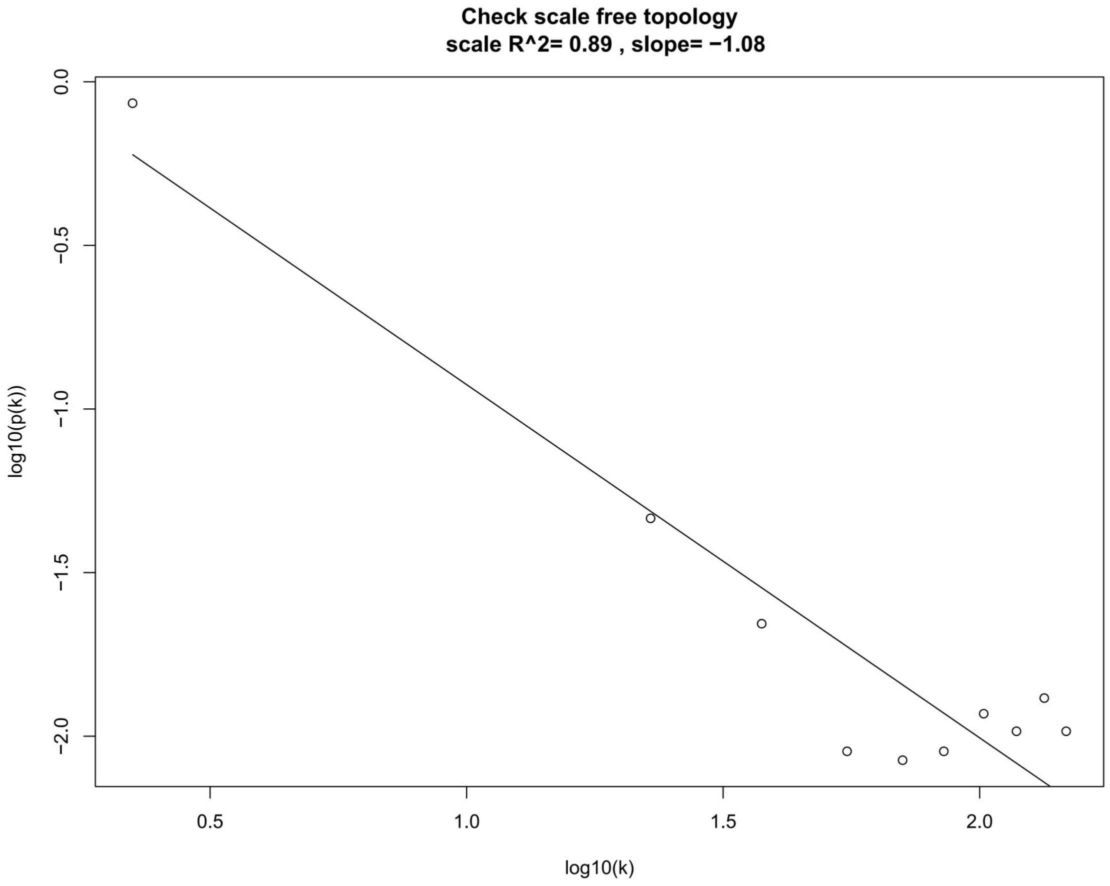

Selecting optimal β parameter for Asthma

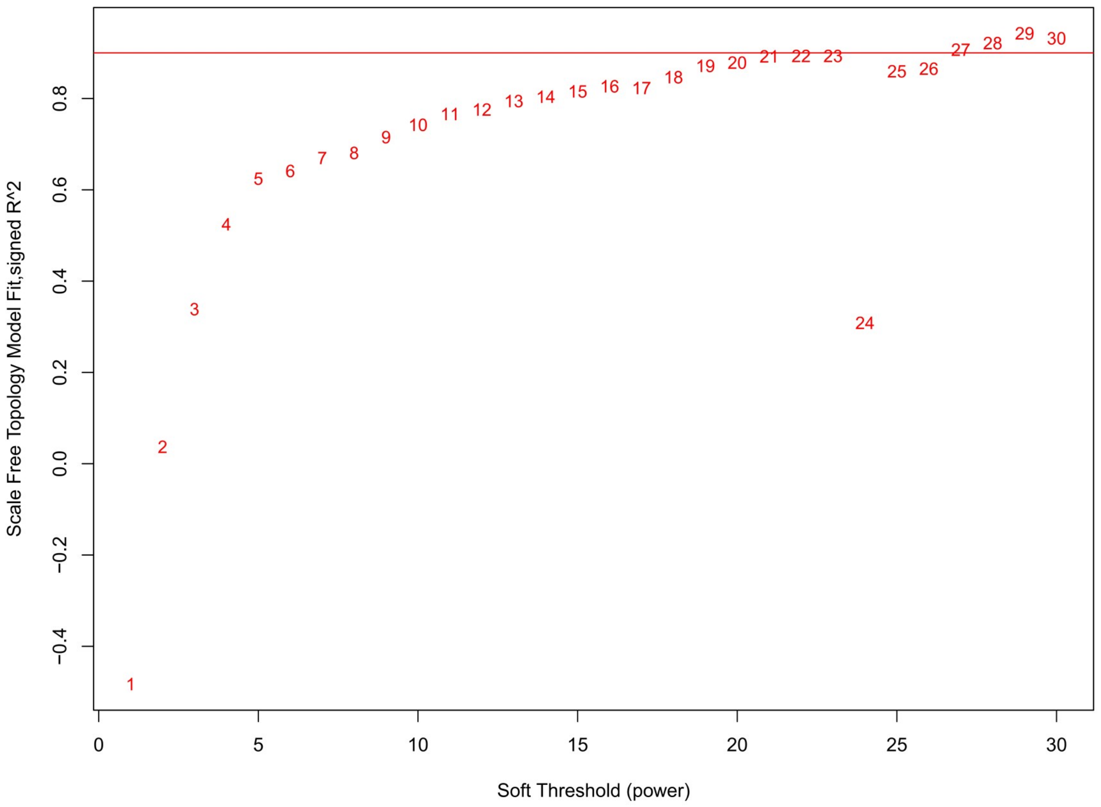

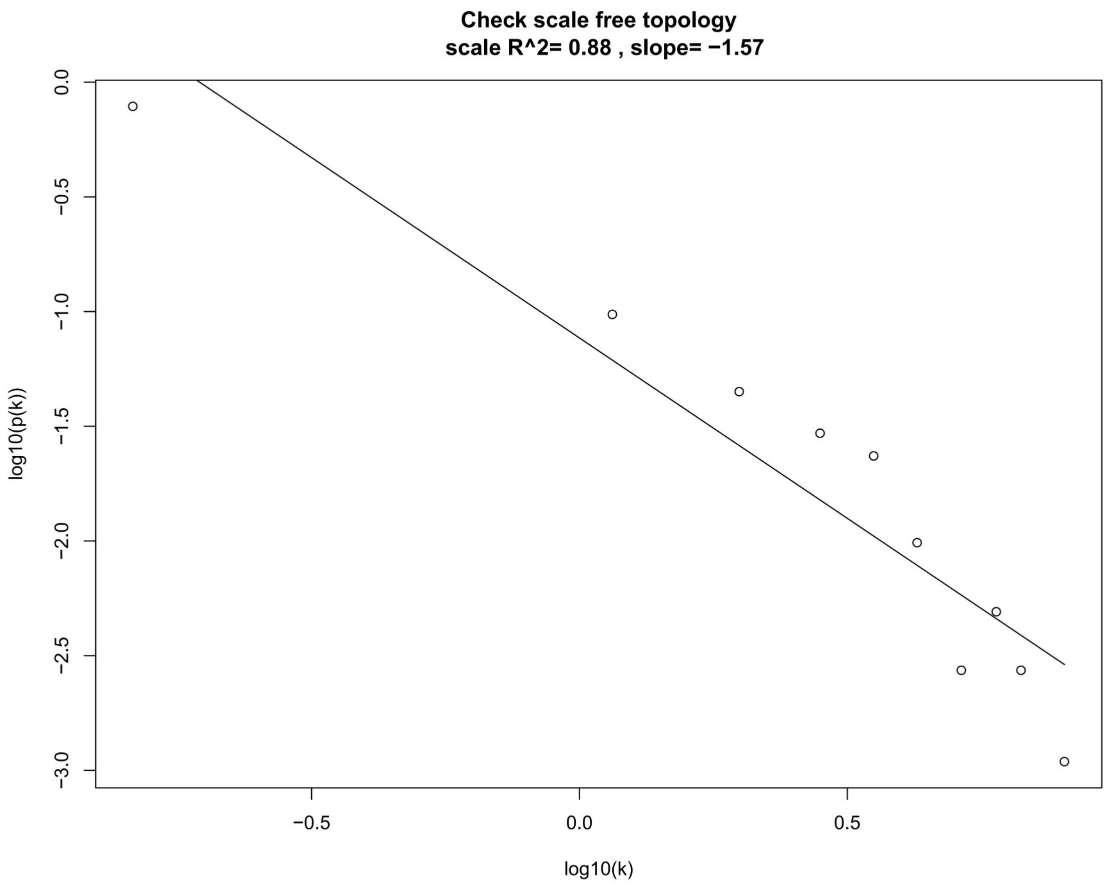

Selecting optimal β parameter for IPF

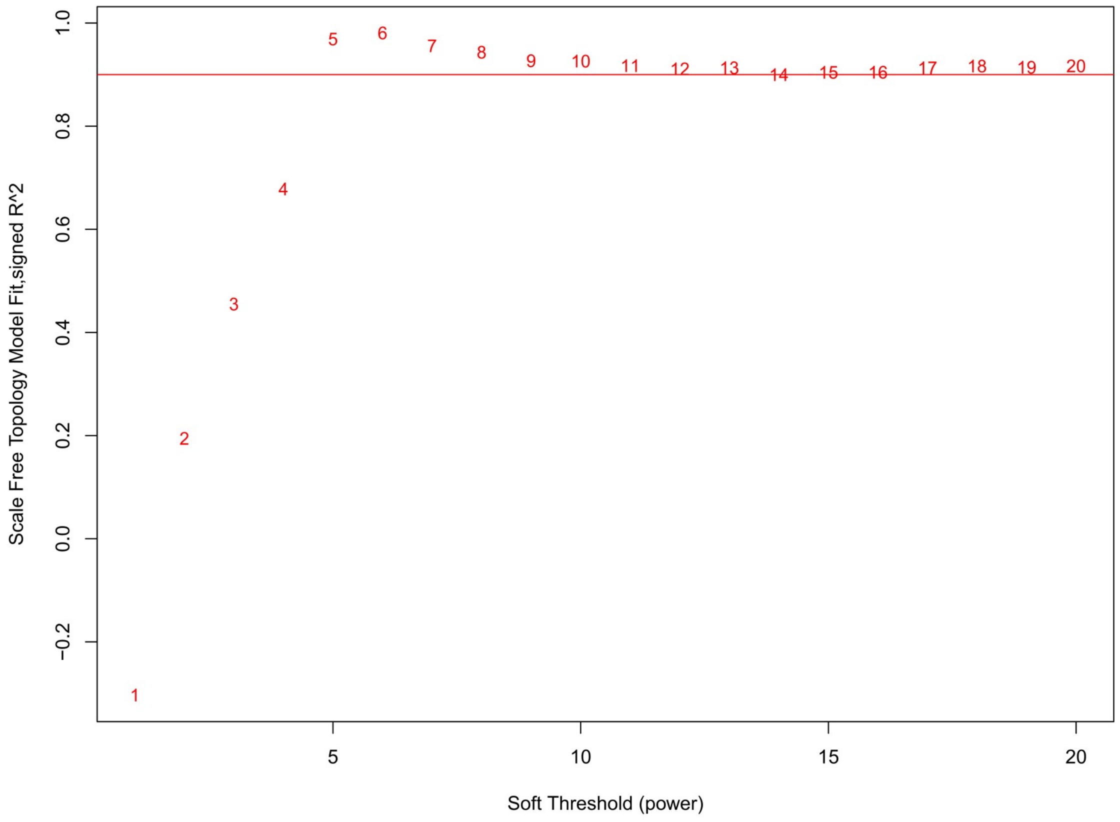

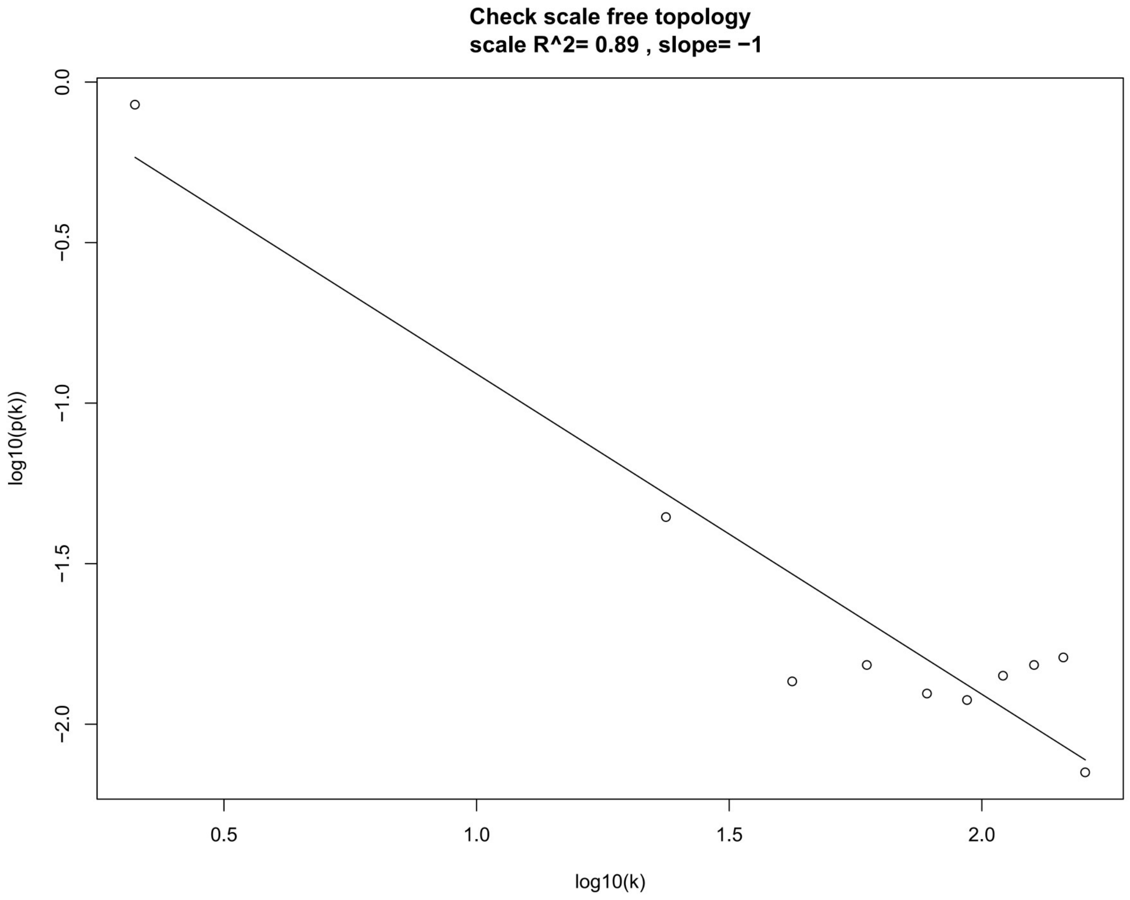

Supplement: Supplementary file 2 — Additional file 2 Selecting the optimal β parameter for disease-specific networks. [file 10020_2019_135_MOESM2_ESM.pdf]
